# Activity Detection from Electricity Consumption and Communication Usage Data for Monitoring Lonely Deaths

**DOI:** 10.3390/s21093016

**Published:** 2021-04-25

**Authors:** Gyubaek Kim, Sanghyun Park

**Affiliations:** 1 Factory Data Development Team, SK Telecom, Seoul 04539, Korea; database.kim@yonsei.ac.kr; 2 Department of Computer Science, Yonsei University, Seoul 03722, Korea

**Keywords:** lonely deaths, safety monitoring, electricity consumption, communication usage, anomaly detection, activity detection

## Abstract

As the number of single-person households grows worldwide, the need to monitor their safety is gradually increasing. Among several approaches developed previously, analyzing the daily lifelog data generated unwittingly, such as electricity consumption or communication usage, has been discussed. However, data analysis methods in the domain are currently based on anomaly detection. This presents accuracy issues and the challenge of securing service reliability. We propose a new analysis method that finds activities such as operation or movement from electricity consumption and communication usage data. This is evidence of safety. As a result, we demonstrate better performance through comparative verification. Ultimately, this study aims to contribute to a more reliable implementation of a service that enables monitoring of lonely deaths.

## 1. Introduction

The death of single-person households has become an increasingly important social problem in many countries. Thus, attempts have been made to provide information communication technology (ICT) based services to alleviate this problem. Most of these services monitor the condition of single persons in real-time through the internet of things (IoT) sensors or smartphone apps [1,2,3,4,5,6,7,8,9,10,11,12,13,14]. Furthermore, the methods of capturing abnormal states are mainstream. Although these methods are real-time-based and widely distributed, they have some limitations. Single-persons who are taken care of by IoT devices feel uncomfortable and find them difficult to operate [15]. This may lead to the discontinuation of the service. Moreover, the total device introduction cost has a large gap with the affordability that the government as a service provider can pay willingly.

For this reason, the use of utility data, such as electricity, water, and gas, has been gaining significant attention [16,17,18,19,20,21,22,23,24,25,26,27,28,29]. This method provides an advantage in that the required data can be easily collected without causing discomfort to single-person households. Usually, it is well prepared to be provided with an open application programming interface (API) by utility companies. This helps the service development cost to be relatively low. In addition, communication data represent another important data source. Activities such as making a call, sending a message, operating smartphone apps, and outing can be found in the data.

However, these data are usually provided one day later. As a result, real-time data analysis is difficult, which is a disadvantage. Considering cost–benefit trade-offs, finding lonely deaths on a daily basis rather than real-time detection is more practical. The latter approach is almost impossible to implement for this purpose. Therefore, solution development using utility and communication data is much more beneficial and promising.

Among all the utility data, the development of solutions using electricity consumption data is preferred. This is probably because of the better readiness compared to other utility data. However, there persist issues with the accuracy of the analysis results. For example, a typical problem is the occurrence of false alarms. In terms of delivering meaningful warnings to a caring agent, it can be useful. However, it needs to be eliminated because it becomes cumbersome for the person in charge.

Electricity consumption and communication history data are all personal information, so privacy is an important issue for this research. Even if it is used for good purposes, such as monitoring lonely deaths, which is an application field of this paper, artificial intelligence (AI) technology should not infringe privacy contrary to its intention. Therefore, the collection of personal information should be minimized, and de-identification should be properly used in the data preprocessing process. By thoroughly complying with the detailed guidelines of the personal information protection act, the possibility of problems can be eliminated.

In this study, we first review existing analysis methods and examine these issues. A new analysis method that infers home appliance activation of single-person households from electricity consumption data is presented to improve the accuracy. This can provide explicit evidence of safety. The process of finding additional activities through communication data analysis is also explained. Furthermore, an experiment using real input and output data from the ongoing service was carried out. Consequently, the proposed analysis method was proven to make more reliable predictions of lonely deaths.

In summary, our contributions are as follows:

1. We propose a new analysis method that recognizes single-person household activities from electricity consumption data. This is mainly different from existing methods using anomaly detection.
2. We propose an additional method to identify activities from communication usage data. It includes explicit operations related to communication services and implicit movements recognized by the analysis of location information.
3. We conduct a performance evaluation of the existing and the proposed methods using real data, compare the result metrics, and prove that our approach shows better performance. This can be referenced in the literature to build more reliable and low-cost monitoring of lonely deaths.

## 2. Related Work

### 2.1. Using IoT Devices

Many services have been provided to monitor single-person households using IoT devices. IoT devices here include video cameras, wearable devices, special-purpose sensors, and smart plugs. The device-based service provision method is summarized as follows:

- Activity detection-based: It intuitively grasps the safety of the person from the data captured by the IoT device [1,2,4,11,12]. Providing an app for a smartphone, as in [13,14], belongs to the method of using an IoT device based on activity detection;
- Anomaly detection-based: Abnormal conditions are analyzed by collecting physical health information (heart rate, electrocardiogram, etc.) [3,5,10].

Some cases use both activity detection and anomaly detection. They first collect activity-related data and then analyze the abnormal state as the next step [6,7,8,9].

### 2.2. Using Utility or Communication Data

**Figure 1:**
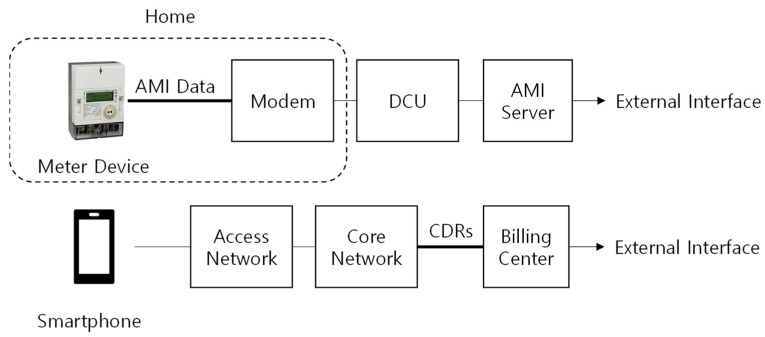
Data generation process in power and communication systems.

There has also been much research on how to use data provided by utility or communication companies. How the data are generated can be seen in Figure 1. Electricity consumption is measured by the meter device, one of the components of the advanced metering infrastructure (AMI). The power line communication (PLC) method is mainly used between the modem installed in the home and the data concentration unit (DCU) outside the home, and in Korea, the introduction rate of such AMI systems currently reaches 43%. Communication usage data are generated in the core network.

The data analysis method was categorized as follows:

- Activity detection-based: It identifies human activities using utility consumption data [16,17,18,19]. Communication usage data processed from call detail records (CDRs) are used to find call patterns and location movements [20,21];
- Anomaly detection-based: It checks whether an abnormality exceeding a specific threshold has occurred. Various techniques such as Bayesian networks, support vector machines, nearest neighbors, clustering, hidden Markov models (HMMs), and neural networks have been applied to detect such anomalies [22,23,24,25,26,27,28,29].

### 2.3. Summary

The data and analysis methods used in the related work can be summarized in Table 1. They are useful for implementing lonely-death monitoring services. However, these methods have both advantages and disadvantages.

As mentioned, despite the advantage of high context awareness, the IoT device utilization method has a problem in terms of introduction cost. Smartphone apps do not cost too much, but there is a disadvantage in that it is inconvenient for single-person households.

**Figure 2:**
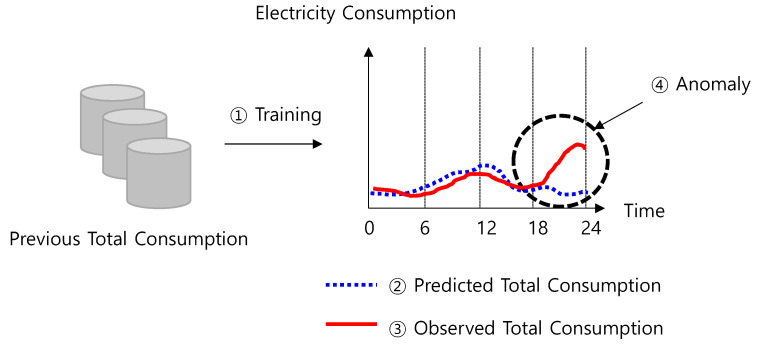
Anomaly detection-based analysis method using machine learning.

Therefore, using utility or communication data has advantages over the method of using IoT devices. The existing approach has mainly adopted anomaly detection-based methods, as shown in Figure 2. This method involves learning from past data, predicting total consumption for the present day, and checking whether the observed consumption is significantly different from the predicted amount. If the difference is too high, it is regarded as an anomaly.

The prediction of total consumption is highly accurate only when dealing with issues of electricity demand across large buildings, cities, and countries. They are composed of many individual consumption points, such as houses, offices, and factories. Because the size of the observation group is large, an error that belongs to different patterns is acceptable within the margin of error. However, the situation is quite different when it is applied to a single household. Predicting electricity consumption, especially for single-person households, may not be successful. This is because it is difficult to infer a person’s lifestyle.

The activity detection-based method using electricity consumption data in the previous work adopted power disaggregation technology, also known as non-intrusive load monitoring (NILM) [30]. However, NILM usually requires 1 s sampled data, and is still limitedly applied to commercialization [31], so it is not feasible to provide a cost-effective lonely-death monitoring service.

Based on prior work, our approach focuses on activity detection using electricity consumption and communication usage data. There have been studies in this direction, but they are not suitable for practical implementation. Therefore, we propose a new pragmatic analytical method.

## 3. Activity Detection

This section introduces the details of our activity-detection method. Our key idea is to use the characteristics of electricity consumption that occur only in single-person households. Communication usage was also assessed. In addition, a method for recognizing movement through location information is proposed.

### 3.1. Analysis of Electricity Consumption Data

The reasoning output of the existing anomaly detection-based analysis method is either “abnormal” or not. As explained, the result in the form of “abnormal” might not be accurate for a few reasons. In contrast, in the proposed new analysis method, the results “normal” or not are presented. If clear evidence of normality is found, it is more appropriate to say “normal” rather than “abnormal”.

**Figure 3:**
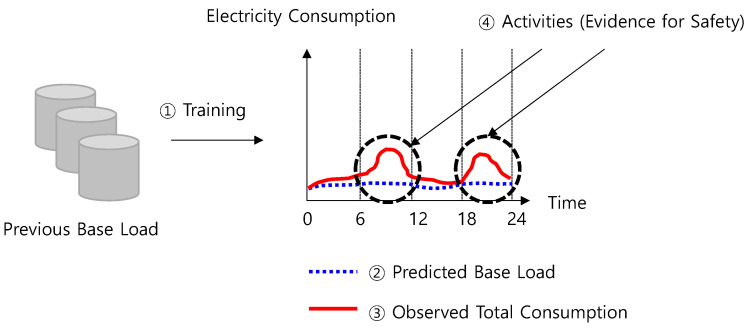
Proposed activity detection-based analysis method.

For this purpose, the base load must be identified. The base load refers to a load generated by constant electricity consumption sources, such as a refrigerator. By extracting the base load from the total electricity consumption, we can identify appliance activation by a single person as clear evidence for safety. The method is shown in Figure 3. This leads to enhanced accuracy in the domain.

#### 3.1.1. Overview

The total electricity consumption (C_T_) measured at home can be defined as the sum of the consumption by appliances (C_A_) that people use and the consumption by the base load (C_B_). The base load is always included in the total electricity consumption, as specified in Equation (1).

C_T_ = C_A_ + C_B_ (1)

It is not possible to know how many and what kinds of appliances constitute the base load, it becomes a factor that makes accurate prediction difficult. Thus, without proper processing of the base load, no analysis model can achieve reliable accuracy.

While monitoring the electricity consumption of single-person households, we discover one important fact that differs from the other average households: if a single person goes out, only the base load (C_B_) occurs in the house. By considering the electricity consumption during the time of going out, the base load can be determined. Hereafter, the base load is used to obtain the activation of the appliance (C_A_) that a single person explicitly makes, and Equation (1) can be rewritten as follows:

C_A_ = C_T_ − C_B_ (2)

In other words, the activation of an appliance at a certain time can be defined by Equation (2). Because the total electricity consumption (C_T_) is measured and provided as raw data for a service, if the base load is obtained by the above logic, the activation of an appliance can be captured.

When C_A_ is close to 0, it means that there is only a base load and no activation. However, if the value of C_A_ is greater than a certain bound, it is assumed that there is an activation. The bound is a tuning parameter that can affect service performance. We focus on the activation of an appliance because it is made by the direct operation of a single-person household, presenting clear evidence of safety.

One interesting aspect of this process is that the base load is not always the same. This is because the composition of the base load can be changed by adding or removing appliances. Moreover, they can show seasonal trends. Therefore, we need to develop a model that trains the base load and predicts it. In our study, the base load was not a simple observation or an average, but it was the predicted result obtained by the analysis model.

#### 3.1.2. Analysis Model

A service maintains each single-person household’s hourly (average) location information. This information can be collected from smartphone apps or location-based services. The latter method is used in this study. As the home address is pre-registered to a service, it is translated into location information in terms of latitude and longitude and compared with the hourly location to obtain the distance. When the distance is greater than a threshold, it is regarded as an outing. As the location information is cell-based, a maximum error of 1 km is used as the threshold in this case.

**Figure 4:**
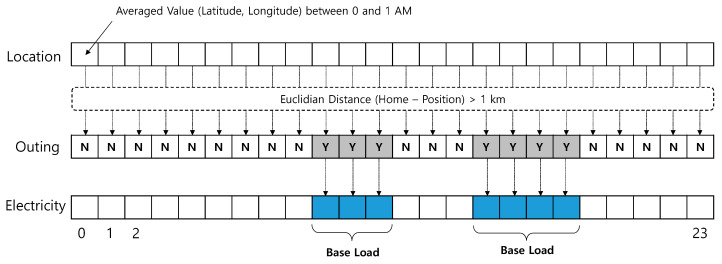
Method of finding base load.

We now have an hourly outing vector through previous processing. This vector is referenced to obtain the corresponding base load. The total steps required to acquire the base load are shown in Figure 4.

**Figure 5:**
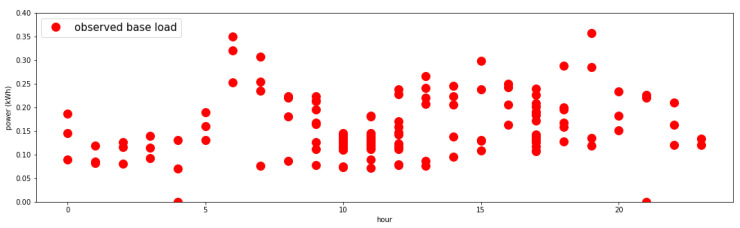
Sample user’s base load by time (raw data).

After iterating the previous step in ascending time order for a certain period (for a month in this study), an aggregated result is collected. Because it is unusual to go out all the time, there are missing values in the time sequence data. The resultant data on a 24 h graph are displayed in Figure 5. The amount of learning data obtained as a result varies depending on each single-person household.

**Figure 6:**
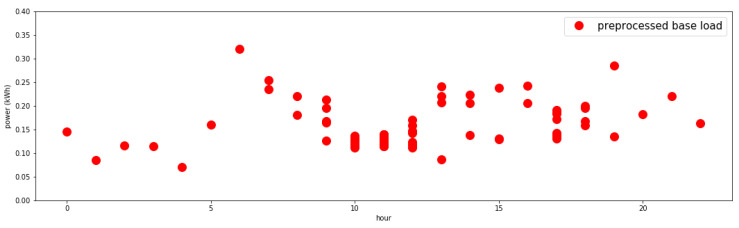
Sample user’s base load by time (pre-processed).

Next, outliers (outside 20% of statistics) are eliminated as part of the pre-processing. The results are shown in Figure 6.

After the base load data for training are prepared, it is possible to develop an analysis model for each single-person household to predict the upcoming base load. The model is re-trained once per month. The training data have missing values, as mentioned. The long short-term memory (LSTM) algorithm is known to be effective in this situation [32] and is hence used to build the analysis model. A detailed summary of the model information that includes some tuned parameters is listed in Table 2. For example, within relatively small epochs, the mean squared error shows up to be 0.0041.

Training data are reshaped into arrays of 36 h data streams. The reason for using a 36 h unit instead of a 24 h unit is to preserve the continuity of time in the input data and prevent incorrect prediction at around 00:00. This is a way to make the analysis model understand that every 00:00 is linked to 23:00 on the previous day. The output from the analysis is also the 36 h unit, but the 24 h unit (00:00–23:00) is the one used daily.

**Figure 7:**
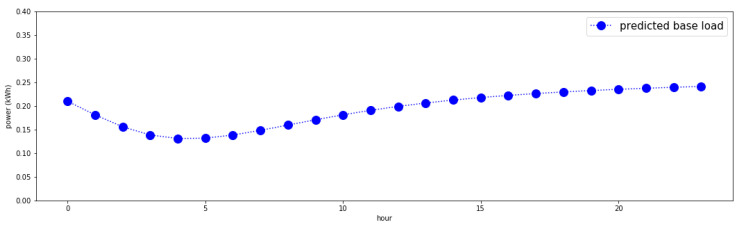
Sample user’s base load by time (predicted).

An example of the predicted base load of a single-person household is shown in Figure 7. It is found that the graph is not just the average or median of that shown in Figure 6; rather, it is a result obtained by reflecting trends in ascending time order. This has the advantage of being able to reflect seasonal changes.

#### 3.1.3. Activity Detection

According to Equation (2), the actual activation of an appliance as evidence for safety can be determined by subtracting the predicted base load from the total electricity consumption.

**Figure 8:**
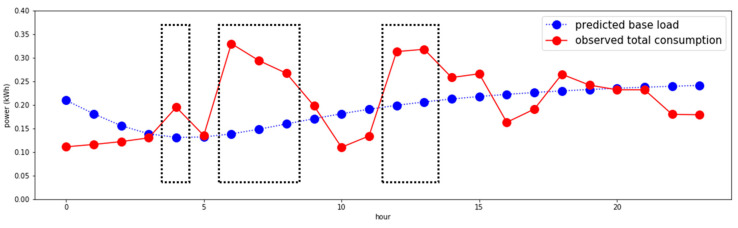
Finding actual positive cases.

The six occurrences (at 4, 6, 7, 8, 12, and 13) surrounded by dotted rectangles reveal clear differences (over 30% of the base load) from the base load in Figure 8. It is concluded that a single-person household is “normal,” if at least one of the positive cases appears during a day.

### 3.2. Analysis of Communication Usage Data

Communication usage data as a life log can be used to check the safety of single-person households. For this, we need to obtain data from a telecommunication company, and consent of the user to provide personal information is required. In general, such data are extracted from CDRs for billing and the information we use is called sending and receiving, text sending, data usage, and location information. Here, text receiving is excluded because it is always accepted regardless of the user’s status.

We analyze the communication data in two ways. First, we simply check whether usage exists or not. Second, we investigate the movement over a very short distance or indoors using location information.

#### 3.2.1. Overview

Checking communication data is straightforward. If a communication history exists, it is regarded as the user’s activity. Whether the user is out is determined by calculating the distance between the home and positioned location.

**Figure 9:**
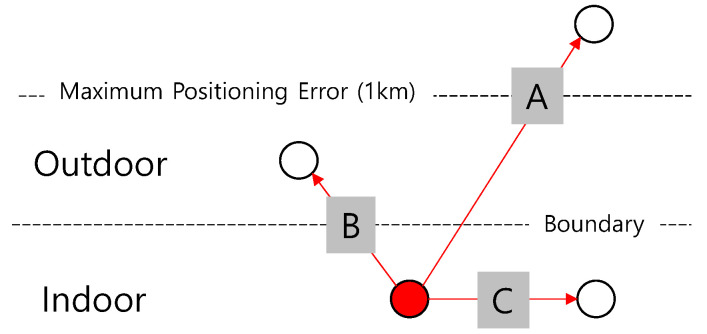
Three cases of user’s movement in our domain.

Next, further examination of the user’s movement is examined. User movement can be divided into three cases, as shown in Figure 9. In the case of A, it is a movement outside the maximum positioning error, which means an obvious outing. Case B usually involves moving a short distance. In the case of C, it represents indoor movement, but it is difficult to recognize because it is movement over a relatively small distance. Therefore, separate indoor positioning technology is required.

Our goal is to find a way to detect the movement in cases B and C without adopting an additional solution. However, if a specific user’s position during the day appears outside the home as only one position on the map, similar to case B, it should not be interpreted as a short distance movement because it may be due to a positioning error.

**Figure 10:**
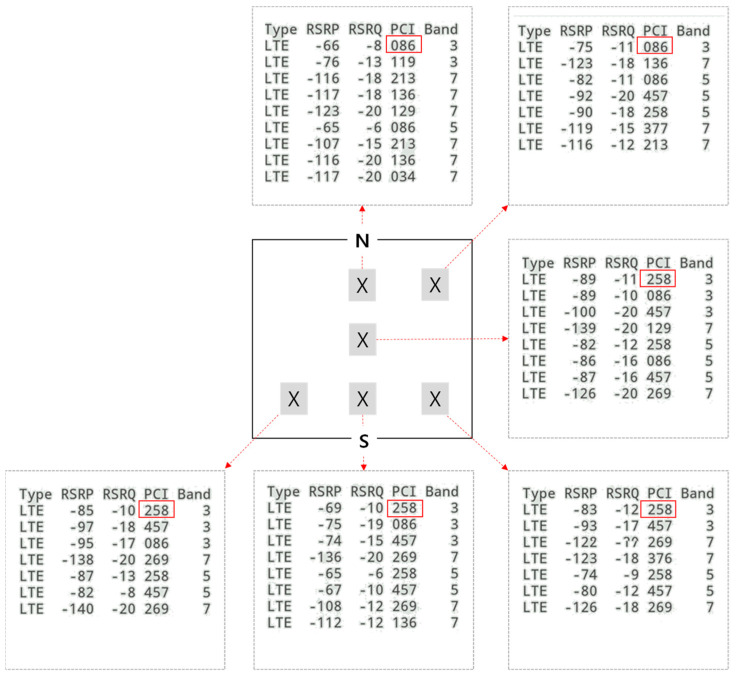
Physical cell IDs (PCI) of neighboring base stations with the strongest signal (measured at author’s house).

In contrast, if more than two positions appear within the maximum positioning error, it can be regarded as a short-distance movement. Figure 10 explains the reasons for this. It shows the base stations that have the highest signal strength at six locations in the sample house. The information is measured by an Android application called LTE Discovery. If we use cell ID-based positioning that only uses the strongest signal base station, it is “086” in the north and “258” in the south. In this case, even if single-person households have been at home all day but stayed in the north and south for a certain amount of time, two different positions are shown on the map.

**Figure 11:**
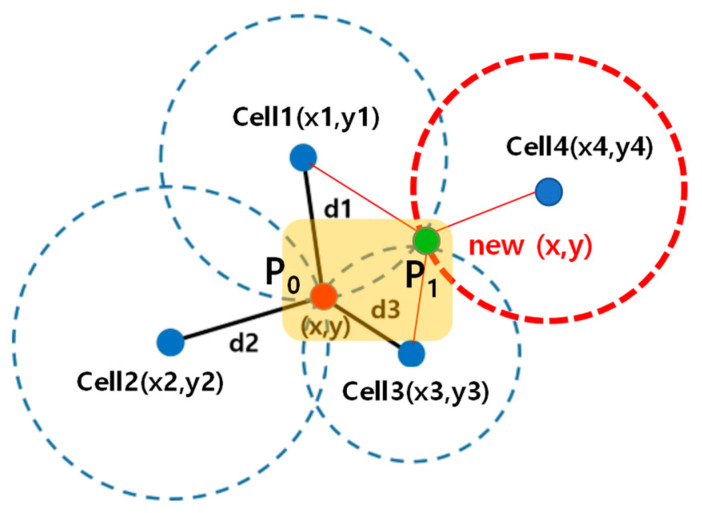
Example movement within a certain bound and the change of positioning result.

A similar result occurs in other positioning methods, such as triangular or primary cell (pCELL), using the signal strength with the base station. Figure 11 shows an example in which the cell list of the strongest signal strength is changed from (1, 2, 3) to (1, 3, 4) according to the user’s movement from left to right within a certain small area. The positioning result was also changed from P_0_ to P_1_ using the triangular positioning method. Similarly, if there is a change in the signal strength of the neighboring base station caused by a small distance movement, positioning results appear at more than two points on the map. Therefore, the short-distance movement of cases B and C can be identified by investigating the distinct number of location clusters on the map.

#### 3.2.2. Analysis Model

The analysis model checks the following conditions to confirm user activity. The first four conditions are collected as raw data, and the last field is calculated using location information, as explained earlier (outing vector in Figure 4). The amount of data usage per hour, 143 KB, is determined by the statistical average survey of single low-income households

**Figure 12:**
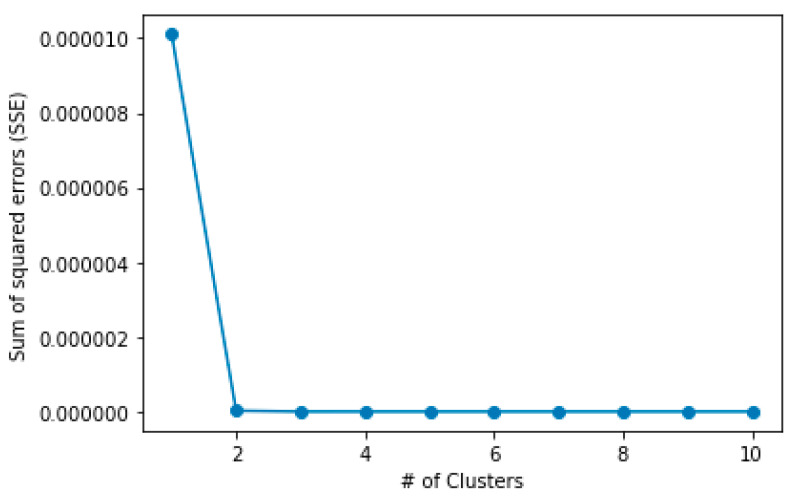
Finding optimal number of clusters using k-means clustering with elbow method.

As explained, in order to determine the movement within the maximum positioning error range, it is necessary to investigate whether two or more positions appear on the map. For this, k-means clustering using the elbow method [33] was used. It helps to find the optimal number of clusters that reduces the sum of squared errors (SSE) the most. Thus, the above example represents the result as two, as shown in Figure 12.

#### 3.2.3. Activity Detection

The activity detection method is performed using communication usage data by executing the following tasks in order:

- First, we check the conditions shown in Table 3;
- Then, we investigate the number of location clusters. If more than two positions appear, we infer that there is a movement within the maximum positioning error.

**Figure 13:**
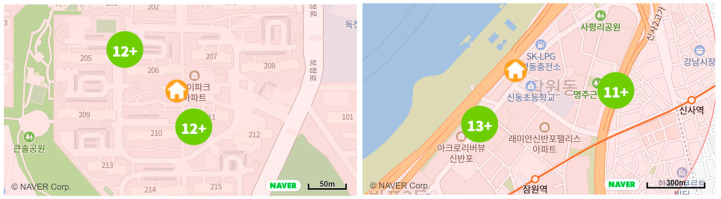
Sample cases of representing two location clusters while staying at home.

Figure 13 shows the results of finding the indoor movement of the sample users. Two different positions as a result of cell ID-based positioning appeared on the map. It was confirmed that users stayed at home all day. Thus, this case represents indoor movement.

### 3.3. Combined Analysis

**Figure 14:**
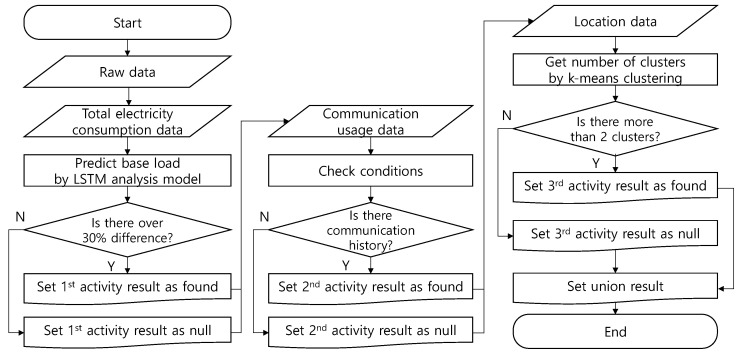
Flow chart of combined analysis.

The two analysis methods described above are not selectively performed, but are integrated into one system. Combining analysis methods such as this increases the probability of finding an activity. In addition, finding multiple pieces of evidence is advantageous because the results can be more reliable. The detailed execution sequence is shown in Figure 14.

## 4. Experiments

To verify the effectiveness of the new analysis method, we compared the existing anomaly detection-based method with the proposed activity detection-based analysis method. For this, we used actual data from real single-person households.

The existing single-person care service that analyzes electricity consumption data only was jointly provided by SK Telecom and Korea Electric Power Corporation (KEPCO). The prediction was made by an autoencoder as one of the deep learning models that periodically learned total electricity consumption data, the only input feature, for approximately a month. The training data had no missing values. It generated a negative prediction when an anomaly was detected. The results obtained by the current service are the baseline for comparison.

### 4.1. Dataset

For the selection of the 88 single-person households that have both electricity consumption and communication usage data, the experiment was conducted for a period of 30 days (16 June 2020–15 July 2020). There was no lonely death during that period. We use a proprietary dataset because public data for benchmarking in this area are not yet available. The experiment was conducted with the data of the user who consented to the provision of personal information.

Unnecessary sensitive information such as social security numbers (SSN) was not collected. This is because there is no problem in implementing the end-to-end service without collecting excessive personal information.

In addition, our service complies with Korea’s personal information protection act similar to general data protection regulation (GDPR). According to the regulation, data that have passed 3 months are strictly deleted.

### 4.2. Evaluation

The estimated result from the new analysis method belongs to one of the following cases in Table 4. Over 50% (51.76 = 24.27 + 24.32 + 2.06 + 1.11) result was confirmed by multiple evidences. This is the key to achieving higher reliability.

**Figure 15:**
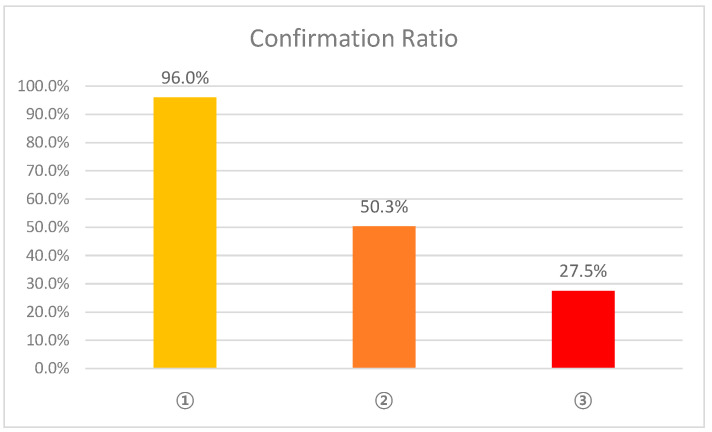
Confirmation ratio of each analysis method.

Each analysis method in the proposed system confirmed actual positive cases with the proportions shown in Figure 15. This represents the degree of impact on the results. For example, 96% of actual positive cases were verified only by the analysis of electricity consumption. Therefore, the analysis of electricity consumption data should be primary and the analysis of communication usage data should be supplementary to the system.

We also examined the analysis results by creating the confusion matrix presented in Table 5. This matrix derives performance metrics, such as accuracy, precision, recall, and f1-score, as defined in Equations (3)–(6).

Accuracy = (TP + TN)/(TP + FP + TN + FN)(3)

Precision = TP/(TP + FP)(4)

Recall = TP/(TP + FN)(5)

F1 Score = 2 × Precision × Recall/(Precision + Recall)(6)

False alarm refers to FN or FP. In general, FP is much more serious. In order to improve the performance of the analysis model, false alarms should be reduced. For this, it is necessary to increase the data to be analyzed or to improve the analysis model algorithm.

### 4.3. Results

**Figure 16:**
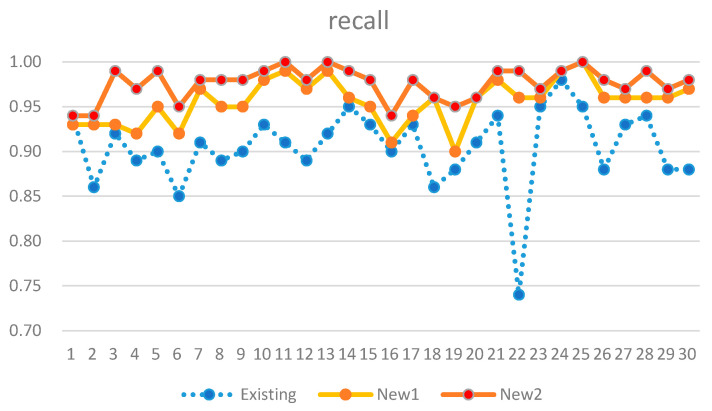
Performance comparison in recall metric.

**Figure 17:**
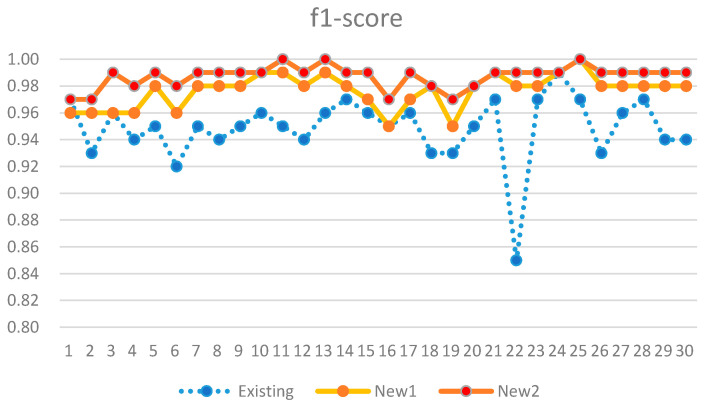
Performance comparison in f1-score metric.

The comparison results of the existing and new analysis methods are listed in Table 6. New 1 uses only activity detection-based analysis from electricity consumption data. In addition, New 2 additionally performs an analysis of communication usage data. In our dataset, there is no actual negative case. Therefore, FP and TN in the confusion matrix are 0. Thus, the precision is always 1. In addition, the accuracy and recall were identical. Therefore, only recall and f1-score are listed in Table 6. The performance metrics of recall and f1-score obtained via the proposed analysis method improved by 8.9% and 4.2%, respectively. Our analysis model only with New 1 showed better performance than the anomaly detection-based existing model by 6.7% in the recall metric. The improvement results are also presented as graphs in Figure 16 and Figure 17.

### 4.4. Discussion

We know the actual positive cases, so TP and FN in the confusion matrix are the most obvious values. Therefore, among all the performance metrics, recall, which is composed of only TP and FN, is a more reliable performance metric. When interpreting the results, recall is considered the most important metric for our purposes. A recall performance close to 1 indicates that unnecessary work is significantly reduced. The possibility of false alarms is 10% in the existing systems. The ratio is sometimes burdensome to the service provider, so improvement is expected.

On 7 July 2020, the existing system suffered from an inaccuracy problem. However, our approach showed stable performance even on the same day.

Our work proved that adding more data to the analysis can improve the performance, rather than using a single source of data. It helped to find more evidence for predicting positive results and could reduce false alarms. Analysis of communication usage data showed an additional 2% improvement in performance.

Therefore, it can be concluded that the proposed method has advantages over the existing anomaly detection-based method. It is more accurate, reliable, and feasible to add more data sources to be analyzed.

The class imbalance issue should be considered, but our dataset has no actual negative cases. Therefore, other performance metrics, such as the FP rate explained in [34], are not given. This is the limitation caused by the dataset used.

## 5. Conclusions

The results of existing analysis methods adopted for our single-person care service are sometimes inaccurate. Hence, it is necessary to develop a new analysis method. This was the motivation for our study. Consequently, unlike existing analysis methods, the proposed analysis method identifies normal activities of single persons.

First, the difference between the total electricity consumption and base load was observed. A large difference clearly indicated that home appliances were activated. Therefore, it was reasonable to accept it as evidence that a single-person household was safe. For this, it was necessary to build an LSTM-based analysis model to predict the base load. A detailed explanation for identifying, learning, and predicting the base load is provided in this paper. Base load often causes inaccuracy in the existing analysis methods, but it is the key to finding more convincing evidence in the proposed analysis method. This activity detection-based analysis showed better performance than the existing anomaly detection-based model.

Moreover, the analysis of communication usage data was also performed using our approach. Only a few single-person households did not use communication in a day, and the data are useful for verification. Explicit activity from communication data was checked. For implicit activity, we additionally introduced an analysis model by investigating location information using k-means clustering to recognize short distance or indoor movement.

The experimental results show that the overall performance of the proposed analysis model is more accurate for all the performance metrics. In particular, the recall metric showed 98% performance.

Based on these results, we plan to replace the existing analysis model in the near future. Furthermore, it is hoped that the proposed technology can be applied to other services such as electronic anklets and self-isolation monitoring. Because these services are also aimed at monitoring special single-person households, the proposed analysis method can be useful for implementation.

## Figures and Tables

**Table 1:** Comparison of methods for implementing care services.

Method	IoT Device	Utility or Communication Data
Activity detection	[1,2,4,11,12,13,14], [6,7,8,9] *	[16,17,18,19,20,21]
Anomaly detection	[3,5,10], [6,7,8,9] *	[22,23,24,25,26,27,28,29]

* Studies that used both analysis methods.

**Table 2:** Base load prediction model.

Parameter	Value
Algorithm	LSTM
Layer	3
Epoch	20
Batch Size	1
Learning Rate	0.001
Train Data Size	0–720 (=24 × 30)
Input Shape	(36, 1)
Average Train Time *	16 s
Elapsed Prediction Time *	<1 s
Mean Squared Error	0.0041
Optimizer	Adam

* Quad core laptop (1.7 GHz CPU and 8 GB memory) is used to train and predict.

**Table 3:** Verifying conditions of communication data.

Condition	Baseline
No. of Inbound Calls	>0
No. of Outbound Calls	>0
No. of Messages Sent	>0
Data Usages Per Hour	>143 KB
No. of Outing	>0

**Table 4:** Cases of evaluated results by proposed method.

① *	② **	③ ***	Proportion (%)
found	found	found	24.27
found	found	-	24.32
found	-	found	2.06
found	-	-	45.37
-	found	found	1.11
-	found	-	0.04
-	-	found	0.15
-	-	-	2.10

* Electricity consumption data analysis, ** Communication usage data analysis (history), *** Communication usage data analysis (movement within a short distance or indoors).

**Table 5:** Confusion matrix and performance metrics.

Cases		Predicted	
		Postive	Negative
Actual	Postive	True Positives (TP)	False Negatives (FN)
	Negative	False Positives (FP)	True Negatives (TN)

**Table 6:** Performance evaluation results.

Date	Existing		New 1		New 2 (Combined)	
	Recall	F1-Score	Recall	F1-Score	Recall	F1-Score
16 June 2020	0.94	0.97	0.93	0.96	0.94	0.97
17 June 2020	0.86	0.93	0.93	0.96	0.94	0.97
18 June 2020	0.92	0.96	0.93	0.96	0.99	0.99
19 June 2020	0.89	0.94	0.92	0.96	0.97	0.98
20 June 2020	0.90	0.95	0.95	0.98	0.99	0.99
21 June 2020	0.85	0.92	0.92	0.96	0.95	0.98
22 June 2020	0.91	0.95	0.97	0.98	0.98	0.99
23 June 2020	0.89	0.94	0.95	0.98	0.98	0.99
24 June 2020	0.90	0.95	0.95	0.98	0.98	0.99
25 June 2020	0.93	0.96	0.98	0.99	0.99	0.99
26 June 2020	0.91	0.95	0.99	0.99	1.00	1.00
27 June 2020	0.89	0.94	0.97	0.98	0.98	0.99
28 June 2020	0.92	0.96	0.99	0.99	1.00	1.00
29 June 2020	0.95	0.97	0.96	0.98	0.99	0.99
30 June 2020	0.93	0.96	0.95	0.97	0.98	0.99
1 July 2020	0.90	0.95	0.91	0.95	0.94	0.97
2 July 2020	0.93	0.96	0.94	0.97	0.98	0.99
3 July 2020	0.86	0.93	0.96	0.98	0.96	0.98
4 July 2020	0.88	0.93	0.90	0.95	0.95	0.97
5 July 2020	0.91	0.95	0.96	0.98	0.96	0.98
6 July 2020	0.94	0.97	0.98	0.99	0.99	0.99
7 July 2020	0.74	0.85	0.96	0.98	0.99	0.99
8 July 2020	0.95	0.97	0.96	0.98	0.97	0.99
9 July 2020	0.98	0.99	0.99	0.99	0.99	0.99
10 July 2020	0.95	0.97	1.00	1.00	1.00	1.00
11 July 2020	0.88	0.93	0.96	0.98	0.98	0.99
12 July 2020	0.93	0.96	0.96	0.98	0.97	0.99
13 July 2020	0.94	0.97	0.96	0.98	0.99	0.99
14 July 2020	0.88	0.94	0.96	0.98	0.97	0.99
15 July 2020	0.88	0.94	0.97	0.98	0.98	0.99
Average	0.90	0.95	0.96	0.98	0.98	0.99

## Data Availability

Data sharing not applicable.
